# Whole-Genome Sequencing of *Hexagrammos otakii* Provides Insights into Its Genomic Characteristics and Population Dynamics

**DOI:** 10.3390/ani15060782

**Published:** 2025-03-10

**Authors:** Dong Liu, Xiaolong Wang, Jifa Lü, Yijing Zhu, Yuxia Jian, Xue Wang, Fengxiang Gao, Li Li, Fawen Hu

**Affiliations:** Laboratory of Benthic Fisheries Aquaculture and Enhancement, Shandong Key Laboratory of Intelligent Marine Ranch (Under Preparation), Marine Science Research Institute of Shandong Province (National Oceanographic Center, Qingdao), Qingdao 266104, China; sdsgld@163.com (D.L.); wxl307@163.com (X.W.); ljifa88@163.com (J.L.); 13791969713@163.com (Y.Z.); jianyuxia79@163.com (Y.J.); 18661733268@163.com (X.W.); 13791940045@163.com (F.G.)

**Keywords:** *Hexagrammos otakii*, K-mer analysis, genome assembly, PSMC, breeding

## Abstract

*Hexagrammos otakii*, also commonly called “Fat Greenling”, is highly valued as an important commercial fish due to its extremely delicious flesh. However, the absence of a genomic resource has limited our understanding of its genetic characteristics and hindered artificial breeding efforts. In this study, we performed Illumina paired-end sequencing of *H. otakii*, generating a total of 73.19 Gb of clean data. Based on K-mer analysis, the genome size was estimated to be 679.23 Mb, with a heterozygosity rate of 0.68% and a repeat sequence proportion of 43.60%. De novo genome assembly using SOAPdenovo2 resulted in a draft genome size of 723.31 Mb, with the longest sequence length being 86.24 Kb. Additionally, the mitochondrial genome was also assembled, which was 16,513 bp in size, with a GC content of 47.20%. Minisatellites were the most abundant tandem repeats in the *H. otakii* genome, followed by microsatellites. In the phylogenetic tree, *H. otakii* was placed within a well-supported clade (bootstrap support = 100%) that included *S. sinica*, *N. coibor*, *L. crocea*, and *C. lucidus*. PSMC analysis revealed that *H. otakii* underwent a population bottleneck during the Pleistocene, peaking around 500 thousand years ago (Kya) and declining to a minimum during the Last Glacial Period (~70–15 Kya), with no significant recovery observed by ~10 Kya. This study was a comprehensive genome survey analysis of *H. otakii*, providing insights into its genomic characteristics and population dynamics.

## Simple Summary

*Hexagrammos otakii*, a commercially valuable cold-water fish, faces threats from overfishing and limited reproductive success. Understanding its genetic characteristics is crucial for developing effective breeding programs and ensuring its long-term sustainability. This study presented the first draft genome of *H. otakii*, providing an essential framework for constructing a high-quality reference genome. The study outlined the genomic characteristics of *H. otakii* and placed it within a robust phylogenetic framework, providing valuable insights for research on related species. The demographic analysis revealed two critical phases in the population history of *H. otakii*, which was crucial for understanding fluctuations in its wild population sizes. As a result, this was the first whole-genome sequencing of *H. otakii* and could be important genomic resource for facilitating future research efforts.

## 1. Introduction

*Hexagrammos otakii*, also known as fat greenling, belongs to the order *Scorpaeniformes*, family *Hexagrammidae*, and genus *Hexagrammos*. This cold-temperate, demersal, reef-associated fish is primarily found along the coasts of the Yellow Sea and Bohai Sea, as well as in the coastal waters of North Korea, Japan, and the Russian Far East [1,2]. *H. otakii* primarily inhabits shallow rocky reef areas, typically at depths of 10 to 30 m. As an omnivorous species, it favors environments abundant in seaweed and rocks, which provide ideal foraging grounds and shelter. This species demonstrates strong tolerance to low temperatures, capable of surviving in water temperatures ranging from 2 to 26 °C, with an optimal growth temperature between 16 and 21 °C. It can adapt to salinity levels ranging from 16 to 32‰. These traits enable it to safely overwinter in northern marine regions [3]. *H. otakii* typically reaches sexual maturity at 2–3 years of age, with mature individuals generally measuring 15–25 cm in length, and males being slightly larger than females. Their breeding season usually occurs from mid-October to late November, when water temperatures drop to around 18 °C. During this period, females lay eggs in rock crevices or seaweed clusters, with the number of eggs ranging from 2000 to 9000 [4].

The meat of the *H. otakii* is tender and flavorful, earning it the nickname “Northern Rockfish”, which makes it an important commercial fish species globally [5,6]. Due to overfishing and environmental changes, the natural resources of *H. otakii* have been nearly depleted. Additionally, female *H. otakii* produce few eggs, which are highly adhesive and tend to clump together, making artificial insemination and incubation extremely challenging. Given the significant reduction in wild populations of this species due to overfishing, habitat loss from coastal development and pollution, a shrinking distribution range, and ongoing threats from environmental pollution and climate change, it is recommended to update its status on the IUCN (International Union for Conservation of Nature) Red List to Near Threatened or Vulnerable [7,8]. These factors have collectively led to increased attention on the genetic characteristics of *H. otakii* to improve artificial breeding techniques, a problem recognized as one of the century’s toughest challenges in academia [9].

Currently, with the development of high-throughput sequencing technologies and the continuous reduction in sequencing costs, significant advancements have been made in studying the genetic characteristics of fish [10,11]. For example, these technologies have enabled researchers to conduct large-scale genome-wide association studies (GWASs) and identify genetic markers associated with important traits such as disease resistance and growth performance [12,13,14]. For *H. otakii*, previous studies have used transcriptomic approaches to investigate the responses of different tissues to environmental changes [15,16]. However, the genomic information for this species remains lacking. This gap in genomic data limits our understanding of its genetic architecture and hinders further advancements in breeding and conservation efforts.

Genomic survey analysis has been proven to be a highly effective and cost-efficient method for rapidly obtaining key genomic characteristics of a study species, such as genome size, heterozygosity rate, repeat sequence proportion, and ploidy level, especially in the absence of a reference genome [17,18]. Using this approach, genomic and evolutionary characteristics have been obtained for multiple fish species [19,20,21]. High-depth clean data generated by it has also provided essential basis for de novo assembly of species genomes. Furthermore, tools like SOAPdenovo2 have demonstrated their effectiveness in generating high-quality fish genomes using paired-end short reads, providing crucial genomic resources for studying genetic traits [22,23,24]. For example, they can be used to accurately characterize the mitochondrial genome and to infer inter-species evolutionary relationships and estimate effective population sizes [25,26,27].

In this study, we conducted the first genome survey analysis of *H. otakii*. Based on high-depth paired-end data, genome characteristics of *H. otakii* such as genome size, heterozygosity, and repetitive sequence proportion were estimated using K-mer analysis. Through genome assembly, we obtained a draft genome and characterized the mitochondrial genome. Satellite DNA sequences were also identified, and the polygenetic relationship with other species was analyzed. Finally, demographic analysis through PSMC (Pairwise Sequentially Markovian Coalescent) revealed significant phases in the population history of *H. otakii*. In summary, our findings might offer new insights into genetic resource conservation and artificial breeding of *H. otakii*.

## 2. Materials and Methods

### 2.1. Specimen Collection and DNA Extraction

The specimens of *H. otakii* utilized in this study were sourced from the breeding base of Marine Science Research Institute of Shandong Province. The individual selected for genome sequencing was identified based on its morphological features, and muscle samples were stored in 95% alcohol for DNA extraction. For the DNA extraction, the conventional phenol/chloroform technique was employed, and RNase A was used to purify the DNA template. The procedure started with the grinding of fish muscle tissue in liquid nitrogen, after which SDS buffer and proteinase K were introduced to disrupt cells and remove impurities. The extraction of DNA was accomplished using a mixture of phenol/chloroform/isoamyl alcohol (25:24:1), and isopropanol was used to precipitate the DNA. The DNA pellet obtained was rinsed with chilled 70% ethanol. Following the evaporation of residual ethanol, the DNA was dissolved in sterile water.

### 2.2. Library Construction, Whole-Genome Sequencing, and Raw Read Quality Control

Genomic DNA extracted from *H. otakii* was used to construct a sequencing library with fragment length of approximately 350 bp. Sequencing was performed on the Illumina NovaSeq 6000 platform (Illumina, SanDiego, CA, USA) with a paired-end read length of 2 × 150 bp, adhering to the manufacturer’s guidelines. FASTP (v0.23.4) software was utilized for the quality control of the raw sequencing data, from which clean data were derived [28]. Quality metrics such as Q20 (the percentage of bases with a quality score over 20) and Q30 (the proportion of bases with a quality score over 30) were calculated to assess the sequencing quality, with a minimum threshold of 90% for both metrics. After removing reads containing adapters, contaminants, and those of low quality, a random selection of 10,000 pairs of clean reads was subjected to BLAST (v2.15.0) analysis against the NCBI Nucleotide (NT) database to identify and filter out any potential contamination [29]. Ultimately, we obtained a high-quality, contamination-free dataset for genome assembly.

### 2.3. Estimation of Genome Size, Heterozygosity Ratio, and Repeat Ratio for H. otakii

To understand the genomic characteristics of *H. otakii*, K-mer analysis was conducted using clean reads to estimate the genome size, heterozygosity, and repeat content. The K-mer size was set to 17, and the K-mer depth distribution was calculated using Jellyfish (v2.2.4) [30]. The 17-mer frequency (depth) distribution was found to be consistent with a Poisson distribution, and the peak depth value was determined, representing the average and variance of the associated Poisson distribution. The genome size of *H. otakii* was estimated using the following equation, G = Kmer-num/Kmer-depth, where Kmer-num is the total number of 17-mers, Kmer-depth is the K-mer depth, and G represents the estimated genome size. This process was facilitated using GenomeScope2 (v2.0) software [31]. Since the K-mer depth distribution can be influenced by heterozygosity and repetitive sequences in the genome, the genome size estimate was revised accordingly. Additionally, the heterozygous frequency and repeat frequency were inferred based on the K-mer analysis.

### 2.4. Sequence Assembly and Analysis of Guanine and Cytosine (GC) Content

Genome sequence assembly was performed using the de Bruijn graph algorithm available in SOAPdenovo (v2.04) with the parameters set as “-d 1 -R -K 127 -p 60” [22]. Contigs were realigned using all clean reads, and scaffolds were constructed step by step using paired-end reads with varied insert sizes. The GC content along the assembled sequence was calculated as the proportion of GC bases out of the total number of bases in assembled sequences. Other assembly metrics, including N50, N90, and max length, were calculated using the Seqkit (v0.16.1) tool [32].

### 2.5. Identification of Simple Sequence Repeats (SSRs)

To identify simple sequence repeat (SSR) markers, SSRs were searched in the assembled scaffolds using the SR search software trf (v4.09) [33]. The search parameters were set to detect di-, tri-, tetra-, penta-, and hexa-nucleotide repeats with a minimum repeat length of 12, respectively. Potential microsatellite motifs were identified using the Perl script “misa.pl” from the MISA (v2.1) software [34].

### 2.6. Mitochondrial Genome Assembly and Annotation

The filtered clean reads were also used to assemble the complete mitochondrial genomes using MitoFinder (v1.4.2) software with default parameters [35]. The annotation of the assembled mitochondrial genome was performed using the Proksee online website [36].

### 2.7. Construction of a Phylogenetic Tree Based on Orthologous Single-Copy Genes

To construct the phylogenetic relationship among *H. otakii*, *Collichthys lucidus* (GCA_004119915.2), *Danio rerio* (GCA_000002035.4), *Larimichthys crocea* (GCA_000972845.2), *Nibea coibor* (GCA_023373845.1), *Oreochromis niloticus* (GCA_001858045.3), and *Sillago sinica* [37], the strategy based on orthologous single-copy genes was employed. Identification of orthologous genes for these species were performed using BUSCO (v5.2.1) with the vertebrata_odb10 database [38] and OrthoFinder2 [39]. This process identified a total of 365 one-to-one orthologous genes across the seven species. Next, protein sequence alignments were conducted using MAFFT (v7.205) [40], and the alignment results of these 365 orthologous protein sequences are available in the Supplementary Materials. Conserved regions of the protein sequence alignments were identified using Gblocks (v0.91b) [41]. These conserved regions were then concatenated to form a supergene. Finally, the species phylogenetic tree using the Maximum Likelihood method was constructed using IQ-TREE v2.2.0 with the parameters “-redo -bb 1000 -mset raxml -m TEST -nt 8” [42].

### 2.8. Inference of Population Size Dynamics for H. otakii

To infer the population size history of *H. otakii*, the Pairwise Sequentially Markovian Coalescent method (PSMC v0.6.5) was employed [43]. Initially, the quality-controlled reads were aligned to the assembled genome using BWA-MEM2 to generate BAM files. Subsequently, the “fq2psmcfa” and “splitfa” tools from the PSMC (v0.6.5) software program were used to prepare the input file for PSMC modeling. PSMC analysis was run with the options -N25 to specify the number of cycles of the algorithm and -t15 to set the upper limit for the most recent common ancestor (TMRCA). The reconstructed population history was visualized using the “psmc_plot.pl” script, with a substitution rate of -u 3.5 × 10^−9^ and a generation time of 0.5 years.

## 3. Results

### 3.1. Genome Survey Analysis of H. otakii

A 350 bp insert library was constructed for Illumina NovaSeq 6000 paired-end sequencing of *H. otakii*. Following adapter trimming and quality filtering, a total of 73.19 Gb clean data, encompassing 494,440,704 reads, was generated (Table 1). Additionally, the results of the NT database comparison showed that the top species matches were related fish species, confirming no significant exogenous contamination during library construction.This dataset exhibited low single-base error rates, with Q20 and Q30 values of 97.5% and 93.23%, respectively, ensuring high-quality data for downstream analyses.

**Figure 1:**
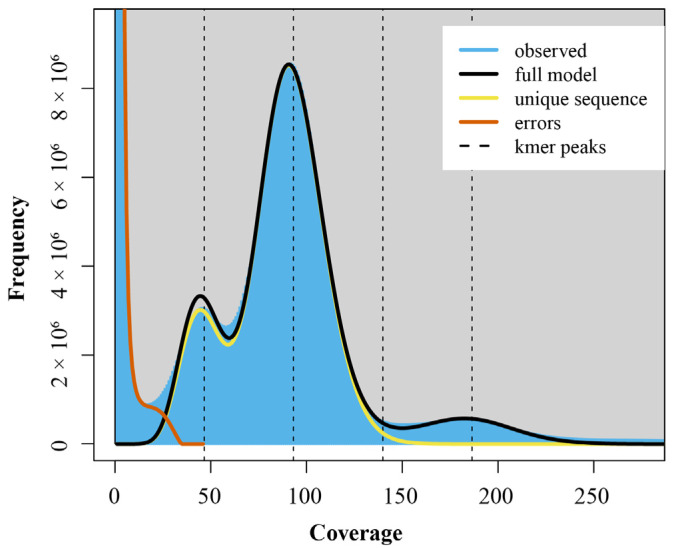
K-mer (K = 17) analysis for estimation of the genomic characteristics of *H. otakii*.

K-mer analysis was used to estimate the genome size, ratio of heterozygosity, and repeats content of *H. otakii*, setting the K-value as 17. The 17-mer frequency plot showed the highest peak at a depth of 97 (Figure 1). The blue area in the graph represents the observed K-mer, and the yellow and orange lines in the graph represent the unique sequences and errors K-mers, respectively.

Then, using the equation genome size = (total number of k-mers)/(the volume peak), the genome size of *H. otakii* was estimated to be 679.23 Mb. The genome exhibited a heterozygosity of 0.68%, and the repetitive sequence proportion was 43.60% (Table 2). These genomic characteristics of *H. otakii* provided crucial insights for the construction of a high-quality, chromosome-level reference genome in subsequent studies.

### 3.2. Genome Assembly and Mitochondrial Genome Assembly of H. otakii

To assemble the draft genome of *H. otakii*, SOAPdenovo2 was used with a specific set of parameter configurations. After the clean data underwent the processes of K-merization, construction of the *De Bruijn* graph, and graph simplification, the raw contig sequences of *H. otakii* were generated (Table 3). A total of 1,484,256 contigs were obtained, with a total length of 716.66 Mb and the longest sequence being 29.26 Kb. Furthermore, the contigs were positioned and oriented, and connected into longer scaffolds using the paired-end information from the sequencing data. The draft genome information of *H. otakii*, which has a size of 723.31 Mb and is composed of 1,224,914 sequences with the longest sequence length being 86.24 Kb and a GC content of 42.40%, was ultimately obtained (Table 3). Compared to the genome size estimated from the 17-mer analysis, the scaffold-level genome assembly had been approximately 44 Mb larger. This discrepancy was primarily attributed to the high heterozygosity rate (0.68%), a phenomenon commonly observed in de novo assemblies of highly heterozygous marine fish species, such as Antarctic notothenioid fish [44]. To address this issue aimed at constructing chromosome-level reference genomes, researchers could have employed third-generation sequencing and Hi-C mapping technologies [4]. These methods would have facilitated the removal of heterozygous sequences using tools like purge_dups [45] and 3d-DNA [46].

**Figure 2:**
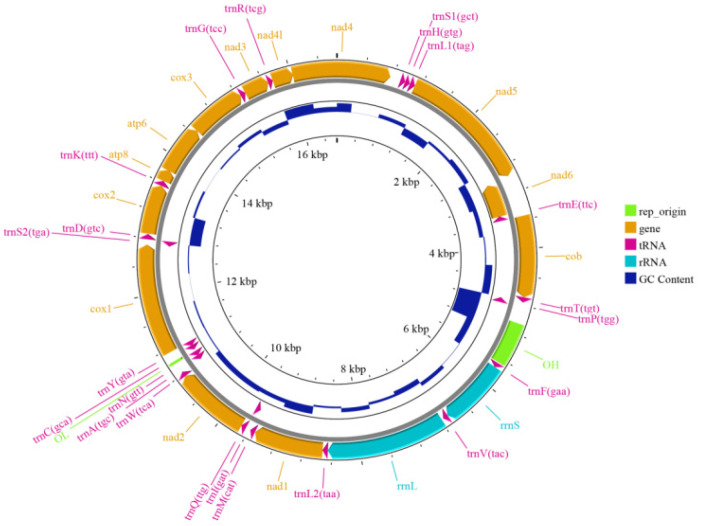
The structure diagram of mitochondrial genomes of *H. otakii*. The inner circle’s dark blue bars represent GC content. The outer circle’s green blocks indicate replication origin regions, yellow arrows represent genes, red arrows represent tRNA, and light blue arrows represent rRNA.

The mitochondrial genome of *H. otakii* was assembled using MitoFinder through processes including read mapping, error correction, graph construction, de novo assembly, and circularization resulted in a circular genome 16,513 bp in length with a GC content of 47.20% (Figure 2 and Table 4).

Upon annotating the mitochondrial genome, we identified a total of 13 protein-coding genes, 22 tRNAs, and 2 rRNAs. Their respective total lengths were 11,427 bp, 1555 bp, and 2614 bp (Table 5). The overall nucleotides base composition of the heavy strand was A (26.90%), G (17.33%), C (29.87%), and T (25.90%). The gene order and composition of *H. otakii* mitochondrial genome was similar to that of most other vertebrates [47]. The mitochondrial genome characteristics of *H. otakii* in our study are entirely consistent with previously reported mitochondrial genome [48], underscoring the reliability of our results.

### 3.3. Profile of Satellite DNA Sequences

Based on the draft genome of *H. otakii*, different types of satellite DNA sequences were identified. In the *H. otakii* genome, three types of satellite sequences were detected: microsatellites, minisatellites, and satellite DNA (Table 6).

Among these, minisatellites, which are repetitive DNA sequences composed of units ranging from 10 to 99 base pairs (bp) in length, were the most abundant. A total of 338,654 minisatellites were identified, with a total length of 48,903,386 bp, accounting for 6.76% of the entire genome. This represented the highest proportion among the three types of satellite sequences. Next, microsatellites, which are repetitive DNA sequences consisting of units that are 1–9 base pairs (bp) long, were also identified. A total of 192,961 microsatellites were found, with a total length of 6,157,332 bp, representing 0.85% of the entire genome. Finally, satellite DNA, which consists of sequences of nucleotides that are at least 100 base pairs long and are repeated in tandem, were identified. A total of 12,649 satellite DNA sequences were detected, with a total length of 3,999,780 bp, accounting for 0.55% of the entire genome. This was the lowest proportion among the three types of satellite sequences (Table 6). In summary, the *H. otakii* genome contains a diverse range of satellite sequences, with minisatellites being the most abundant, followed by microsatellites, and then satellite DNA. These repetitive elements play important roles in genome structure and function. These results may facilitate the development for species identification molecular makers and further contribute to the population genetics of *H. otakii.*

### 3.4. Phylogenetic Relationship of H. otakii with Six Other Species Based on Orthologous Genes

**Figure 3:**
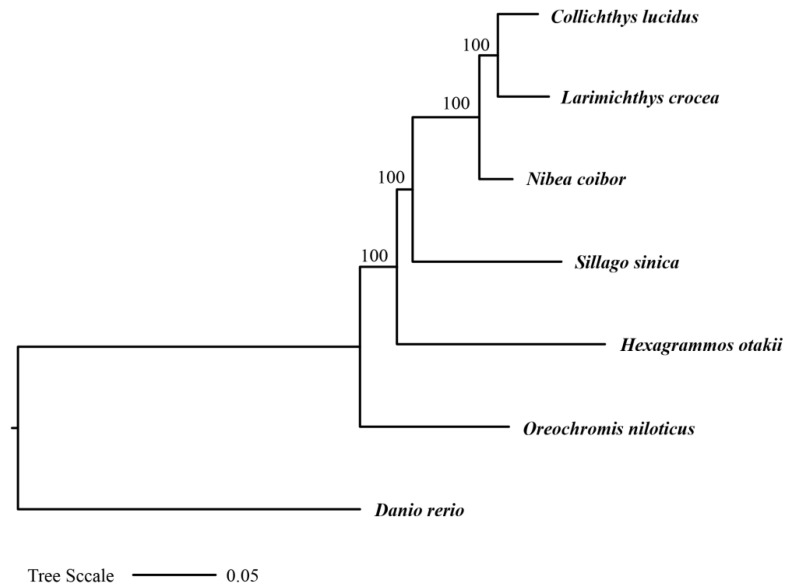
The ML phylogenetic tree inferred from the 365 one-to-one orthologous genes of seven species.

To investigate the evolutionary position of *H. otakii* within the order *Perciformes*, we constructed a phylogenetic tree including *H. otakii* and five other species from different genera within *Perciformes*: *Oreochromis niloticus*, *Sillago sinica*, *Nibea coibor*, *Larimichthys crocea*, and *Collichthys lucidus*. Additionally, *Danio rerio* from the order *Cypriniformes* was used as an outgroup. A total of 365 one-to-one orthologous genes across the seven species were identified, and the phylogenetic relationship was constructed using IQ-TREE v2.2.0 (Figure 3). The tree was rooted, and branch support was assessed using 1000 bootstrap replicates.

In the phylogenetic tree, *H. otakii* was placed within a well-supported clade (bootstrap support = 100%) that included *S. sinica*, *N. coibor*, *L. crocea*, and *C. lucidus*. The high bootstrap support for these relationships added credibility to the inferred evolutionary relationships, highlighting the robustness of the phylogenetic analysis.

### 3.5. The Population Size Dynamics of H. otakii

**Figure 4:**
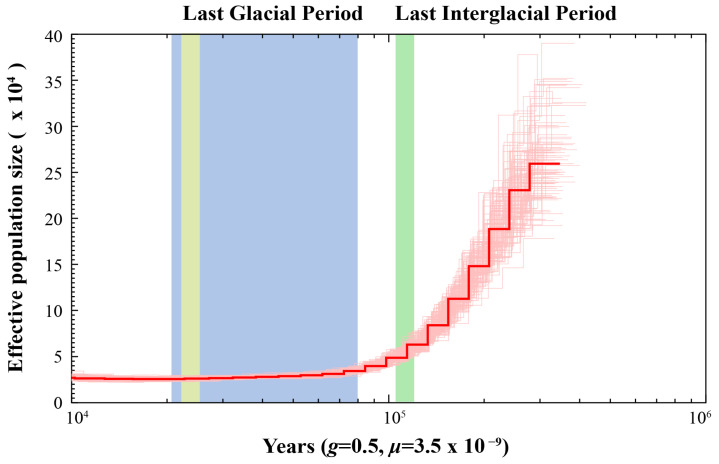
Effective population size estimates of *H. otakii*. The *x*-axis represents the time before present, ranging from 10 thousand years to 1000 thousand years (Kya) from left to right. The *y*-axis represents the effective population size. The parameter *g* denotes the generation time of the species, measured in years, and *μ* represents the mutation rate of the species.

PSMC analysis revealed that *H. otakii* underwent a population bottleneck during the past million years, with its effective population size peaking around 500 thousand years ago (Kya) before beginning to decrease (Figure 4). During the Last Interglacial Period (~130–116 Kya), the effective population size of *H. otakii* decreased at a constant rate from its peak. *H. otakii* primarily inhabits cold-water regions of the North Pacific, typically at depths between 10 and 150 m [5]. During the Last Interglacial, global temperatures rose, sea levels increased, and glaciers melted. The sustained decline in population size during this period may have been driven by changes in habitat, such as the loss of shallow coastal areas, and alterations in ecosystem structure, which made it difficult for the species to maintain a large population [1]. Eventually, the effective population size of *H. otakii* reached a minimum during the Last Glacial Period (~70–15 Kya), with no observable trend of recovery by ~10 Kya. During the Last Glacial Period, harsh environmental conditions likely led to a further contraction of habitats, scarcity of food resources, and population fragmentation, ultimately resulting in a significant reduction in population size. Additionally, lower sea levels during this period may have severed certain migration routes, limiting gene flow between populations [49,50]. Taken together, these results suggest that *H. otakii* experienced a bottleneck effect during the Pleistocene Glacial Epoch.

## 4. Discussion

This is a comprehensive genome survey analysis of *H. otakii*. In this study, we generated a total of 73.19 Gb of clean data, with a genome size estimated at 679.23 Mb based on K-mer analysis, achieving a sequencing depth of over 107×. Both the Q20 and Q30 values of the sequencing data exceeded 90%, demonstrating the high depth and quality of our data. High-depth and high-quality sequencing data are essential for subsequent genomic analyses, as multiple previous studies have shown [51,52,53]. Using high-quality data, we performed de novo assembly to generate the first draft genome of *H. otakii*, resulting in a genome size of 723.13 Mb, which is larger than what was predicted by K-mer analysis. This size discrepancy may stem from the elevated heterozygosity rate of *H. otakii*. Comparable findings in other species support this theory, demonstrating that bioinformatics tools can effectively distinguish heterozygous sequences, thereby reducing redundancy in genome assemblies [54,55].

Mitochondria play a crucial role in the biological functions of fish, involving multiple aspects such as energy metabolism, cell apoptosis, genetic diversity, and evolutionary studies [56,57]. Therefore, characterizing the mitochondrial genome of *H. otakii* was essential. In our study, we identified the mitochondrial genome of *H. otakii* to be 16,513 bp long, with a GC content of 47.20%, and it contains 22 protein-coding genes, which is consistent with previous studies [47,48]. The majority of the genes were encoded on the heavy strand, whereas the NADH dehydrogenase subunit 6 (ND6) and eight tRNA genes [Gln, Ala, Asn, Cys, Trp, Glu, Pro, and Ser (TGA)] are encoded on the light strand. The heavy strand’s nucleotide base composition is 26.90% A, 17.33% G, 29.87% C, and 25.90% T, showing a bias toward A + T. This consistency further validated the reliability of our assembly results.

The PSMC method for estimating effective population size has had significant applications in fish genetics research, aiding in understanding species history and evolution, and providing crucial scientific insights for conservation, breeding, and ecological studies [58,59]. Our analysis revealed that the effective population size of *H. otakii* underwent two distinct periods: a rapid decline between 1,000,000 and 10,000,000 years ago, followed by a period of stabilization between 100,000 and 1,000,000 years ago. The period of decline suggested that *H. otakii* experienced a significant reduction in population size, possibly due to environmental changes such as climatic shifts or habitat loss, or other external pressures like predation or competition [60,61]. However, the stabilization of the effective population size indicated that the population reached a new equilibrium after the initial decline, likely due to adaptation to new environmental conditions or the recovery of the population from the previous bottleneck [60,61].

Although our study has provided a high-quality genomic scaffold of *H. otakii*, the development of whole-genome sequencing technologies has made it possible to generate chromosome-level, telomere-to-telomere (T2T), and even gap-free reference genomes for an increasing number of fish species [62,63,64]. Integrating short reads sequencing, long reads sequencing, and Hi-C mapping technologies could yield a high-quality chromosome-scale reference genome of *H. otakii*, with a genome size of 682.43 Mb, a contig N50 size of 2.39 Mb, and a scaffold N50 size of 27.83 Mb [4]. While our study mainly focused on comprehensively investigating the genomic characteristics, evolutionary relationships, and population dynamics of *H. otakii*. This would offer a vital genetic resource for important traits enhancement, selective breeding, and aquaculture practices for *H. otakii*.

## 5. Conclusions

This study presented the first whole-genome survey and de novo assembly of *H. otakii*, which provided a foundation for the subsequent high-quality genome construction. The genome size was estimated to be 679.23 Mb, exhibiting a heterozygosity rate of 0.68% and a repeat sequence proportion of 43.60%. De novo assembly yielded a genome size of 723.31 Mb. The mitochondrial genome was 16,513 bp with a GC content of 47.20%. Minisatellites were the most abundant satellite DNA, followed by microsatellites, in the *H. otakii* genome. Furthermore, the phylogenetic analysis placed *H. otakii* within a well-supported clade, and PSMC analysis revealed *H. otakii* experienced a bottleneck effect during the Pleistocene Glacial Epoch. This important genomic resource will significantly advance our understanding of *H. otakii*’s genetic characteristics and facilitate future research efforts, including genetic improvement programs and a deeper exploration of its evolutionary history.

## Figures and Tables

**Table 1:** Statistics of next-generation sequencing data.

Species	Read Number	Base Count (bp)	Read Length (bp)	Q20 (%)	Q30 (%)
*H. otakii*	494,440,704	73,193,542,644	150	97.50	93.23

**Table 2:** K-mer analysis of *H. otakii* genome.

Metrics	Value
*K-mer* size	17
*K-mer* depth	97
Genome size (bp)	679,232,407
Genome repeat length (bp)	296,145,329
Genome unique length (bp)	383,087,078
Repeat (%)	43.60%
Heterozygous ratio (%)	0.68%

**Table 3:** Statistics of genome assembly of *H. otakii* genome.

Assembly Level	Total Length (bp)	Total Number	Max Length (bp)	N50 Length (bp)	N90 Length (bp)	GC Content (%)
Contig	716,659,970	1,484,256	29,264	695	230	42.79
Scaffold	723,306,185	1,224,914	86,243	1183	246	42.40

**Table 4:** Assembly of the *H. otakii* mitochondrial genome.

Assembly Level	Total Length (bp)	GC Content (%)
Contig	16,513	47.20

**Table 5:** Annotation of the mitochondrial genome of *H. otakii*.

Type	Total Number	Total Length (bp)	Mean Length (bp)
Gene	13	11,427	879
tRNA	22	1555	71
rRNA	2	2614	1307

**Table 6:** Statistic of different types of satellite DNA in *H. otakii* genome.

Type	Definition	Total Number	Total Length (bp)	Rate (%)
Microsatellite	1–9 bp units	192,961	6,157,332	0.85
Minisatellite	10–99 bp units	338,654	48,903,386	6.76
Satellite	≥100 bp units	12,649	3,999,780	0.55
Total		544,264	59,060,498	8.17

## Data Availability

The Illumina paired-end reads have been deposited in the Genome Sequence Archive (GSA:CRA021610, https://ngdc.cncb.ac.cn/gsa, accessed on 20 January 2025).

## Supplementary Materials

The following supporting information can be downloaded at: https://www.mdpi.com/article/10.3390/ani15060782/s1, Supplementary Data S1: Species_Orthologs_supergene_pep.fasta; Supplementary Data S2: Species_Orthologs_supergene_pep.phy.
